# THC degradation does not impair the accuracy of THC doses aerosolized by the metered-dose SyqeAir inhaler: a 24-month stability trial

**DOI:** 10.1186/s42238-022-00166-5

**Published:** 2022-10-24

**Authors:** Joshua Aviram, Daniella Atzmony, Anna Frenklakh, Asaf Kroll, Ilana Zaks, Arno Hazekamp

**Affiliations:** 1 Syqe Medical Ltd, 14 Hatchiya Street, Studio 211, 6816914 Tel Aviv-Yafo, Israel; 2 Hazekamp Herbal Consulting, Leiden, The Netherlands

**Keywords:** Phytocannabinoids, Medical cannabis, Stability, Inhalation

## Abstract

**Background:**

Although the worldwide use of medical cannabis (MC) is on the rise, there is insufficient data regarding the long-term stability of phytocannabinoids in the plant material under different storage conditions. Specifically, there is insufficient data on the effect of storage conditions on the availability of (-)-∆^9^-trans-tetrahydrocannabinol (THC) in vaporized cannabis. The Syqe inhaler delivers metered doses of phytocannabinoids by inhalation and utilizes accurate quantities of ground cannabis inflorescence packaged in tamper-proof cartridges. We aimed to assess the stability of phytocannabinoids in ground cannabis before and after packaging in Syqe cartridges as well as the reproducibility of THC delivery in the aerosolized dose.

**Methods:**

Ground MC inflorescence was stored under different temperature and humidity conditions, before or after being packaged in Syqe cartridges. Concentrations of the major phytocannabinoids therein were analyzed at different time points using ultra-high performance liquid chromatography (U-HPLC). THC doses aerosolized via the Syqe inhaler were evaluated using cartridges stored for up to 2 years at 25°C. Every vapor chip contains 13.5±0.9 mg of ground MC powder.

**Results:**

No significant changes were observed in phytocannabinoid concentrations in ground cannabis inflorescence after 3 months of bulk storage in a polypropylene container and sealed in an aluminum foil pouch at 5°C. In contrast, significant changes in phytocannabinoid concentrations were found when ground inflorescence was stored in the cartridges at 25°C for 2 years. Specifically, CBGA, THCA, and total THC concentrations decreased from 0.097±0.023, 2.7±0.3, and 2.80±0.16 mg/chip at baseline to 0.044±0.007 (55% decrease), 1.50±0.27 (44% decrease), and 2.20±0.083 (21% decrease) mg/chip following 2 years, respectively, while CBN and THC concentrations increased from 0.005±0.005 and 0.44±0.11 mg/chip at baseline to 0.14±0.006 (2700% increase) and 0.88±0.22 (100% increase) mg/chip following 2 years, respectively. Storage at 30°C revealed a steeper change in phytocannabinoid concentrations within an even shorter period. Despite the significant change of relative cannabinoid composition within the cartridge, the actual THC dose present in the aerosol remained relatively stable throughout this period and within the dosage range of 500mcg±25% required for pharmaceutical-grade inhalers.

**Conclusions:**

MC powder in Syqe cartridges may be stored at room temperature for at least 2 years after production without affecting the aerosolized THC dose delivered to patients by more than ±25%. Future studies should analyze additional phytocannabinoids and terpenes in the cannabis inflorescence and assess the stability of different cannabis cultivars following storage in Syqe cartridges.

## Introduction

The interest of Western modern medicine in the therapeutic potential of *Cannabis sativa* L. (*Cannabis*) started following the report of O’Shaughnessy in his 1839 book on the therapeutic effects of Indian hemp (O’Shaughnessy, 1839). Since then, despite a long period of *Cannabis* prohibition (David et al., 2014), treatment of many clinical indications using medical cannabis (MC) has been on the rise (Boehnke et al, 2016). In recent decades, research on *Cannabis* has significantly advanced and, thus far, 144 phytocannabinoids were identified (Berman et al, 2018). The main natural phytocannabinoids are (-)-∆^9^-trans-tetrahydrocannabinolic acid (THCA), cannabidiolic acid (CBDA), and cannabigerolic acid (CBGA). As these compounds contain a chemically unstable carboxyl group (COOH), when heat is applied they are converted by a process known as decarboxylation into (-)-∆^9^-trans-tetrahydrocannabinol (THC), cannabidiol (CBD), and cannabigerol (CBG), respectively (Peschel, 2016).

Another compound, cannabinol (CBN), which is formed by THC oxidation and by cannabinolic acid (CBNA) decarboxylation, has been established as a marker for *Cannabis* aging.

*Cannabis* cultivars are typically classified by their THC and CBD concentrations (Peschel, 2016). THC-rich cultivars are designated as type I, CBD-rich cultivars as type III, and cultivars with comparable concentrations of THC and CBD as type II (Russo, 2011). This categorization system is currently the basis of most clinical treatments using the *Cannabis* plant (Aviram et al, 2020a) in countries where MC use has been legalized.

MC is mostly administered via smoking or vaporization (Hazekamp et al, 2013; Aviram et al, 2020b) but these administration routes are not metered or accurate (Huestis, 2009). To overcome the problem of inaccurate dosing, the Syqe metered dose inhaler (Trade name: SyqeAir inhaler, Syqe Medical Ltd., Tel Aviv, Israel) was developed. The inhaler is a battery-operated, hand-held, thermal-selective metered-dose inhaler that utilizes a tamper-proof cartridge containing vapor chips (VCs). Each VC holds a precise quantity of processed medical-grade *Cannabis* powder from dried natural *Cannabis* inflorescences manufactured under cleanroom conditions (Lai et al, 2005). The inhaler is preprogrammed with several distinct heating protocols, each tailored to heat a VC and deliver an aerosol containing 250, 500, 750, or 1000 mcg of THC to the patient. The inhaler’s settings can be selected at the press of a button. In operation, the inhaler heats the MC to a temperature below combustion and engages automatic thermal and airflow controls that ensure precise, accurate, and high-efficiency delivery of the selected dose of MC aerosol to the patient’s lungs, independent of the patients’ inhalation pattern. The patient has no direct contact with the MC, and only inhales phytocannabinoids vaporized there from. The device requires minimal training prior to use and automatically generates logs of the patient treatment regimen (dose × time of day) that connects to an app on the patient smartphone, so that the patient as well as the treating physician can have information regarding the patient’s actual adherence to a prescribed regimen and thereby learn to optimize the treatment over time.

Currently, THC is used for dose selection (e.g., 500 mcg THC) and it serves as an indicator for the amounts of other molecules, such as minor phytocannabinoids, terpenes, and more that vaporize into the aerosol during the vaporization of a selected THC dose. In recent years, Syqe inhaler technology was evaluated in several clinical trials (Eisenberg et al, 2014; Vulfsons et al, 2020; Almog et al, 2020) and in a long-term observational study (Aviram et al, 2022), where it demonstrated effectiveness, safety, and usability as well as narrow pharmacokinetic variability.

Milay et al. (2020) investigated the optimal storage conditions for preserving the composition of naturally occurring secondary metabolites (i.e., phytocannabinoids and terpenoids) in *Cannabis* inflorescences and extracts postharvest over a 1-year period. They reported significant variability in the stability of the main phytocannabinoids at room temperature (25°C) and concluded that the best conditions for preserving the original phytocannabinoid and terpene contents of inflorescences throughout long storage periods are in the form of whole (non-ground) inflorescences at 4°C (Milay et al, 2020). In the current study we aimed to assess the stability of these phytocannabinoids during the manufacturing process (as will be described in the Methods section) at the Syqe Medical facility and within the vacuum-sealed cartridges for a period of 2 years. In addition, we assessed whether storage time has an effect on the dose of THC aerosolized via the Syqe inhaler from such cartridges. In this stability study, only the 500 mcg THC dose was assessed as it was found to be the optimal dose for chronic pain treatment in a previous study, with the best balance between pain intensity reduction report and intoxication levels (Almog et al, 2020).

## Methods

### Chemicals and reagents

Liquid chromatography LiChrosolv® gradient grade acetonitrile, methanol, and water for the mobile phase were purchased from Mercury Scientific and Industrial Products Ltd. (Rosh Haayin, Israel). Liquid chromatography-mass spectrometry (LC-MS)-grade formic acid was purchased from BioLab Ltd. (Jerusalem, Israel). Phytocannabinoids analytical standards (>98% purity) of THC, THCA, CBD, CBDA, CBN, and CBGA were manufactured by Restek (Bellefonte, PA, USA). Data were restricted to the requirements of the Israeli Medical Cannabis Authority regulations.

### Medical cannabis

The MC inflorescence product “Bedrocan” (Bedrocan International, Veendam, Netherlands), which contains about 22% THC and <1.0 CBD, was used in all experiments. It was free of pesticides, heavy metals (<0.2 ppm lead, <0.02 ppm mercury, and <0.02 ppm cadmium), stalks, and foreign materials. Microbiological purity was also confirmed by the manufacturer (total aerobic microbial count of <10 colony forming units [CFU]/g, total yeast and mold count of <10 CFU/g, and absence of *Pseudomonas aeruginosa*, *Staphylococcus aureus*, and bile-tolerant gram-negative bacteria).

### MC processing, cartridge production, handling, and storage

Before processing, the MC powder is stored at 25°C (± 2°C) and relative humidity (RH) of 60% (± 5%) in a polypropylene container, sealed in an aluminum foil pouch (Oliver-Tolas Healthcare Packaging B.V., Venray, the Netherlands). All MC handling — starting from processing until a cartridge is vacuum sealed — are performed in a clean-room environment. Manufacture begins with the grinding of whole *Cannabis* inflorescences (Bedrocan MC inflorescence product) to form a fine and homogenized powder (particle diameter <1000 μm).

VCs are manufactured by a dedicated machine, which allots 13.5±0.9 mg of ground MC powder into each VC. Sixty VCs are subsequently assembled into each tamper-proof cartridge which is then packaged in a vacuumed aluminum foil that protects it from exposure to light and oxygen until use. The Syqe inhaler releases a single VC for each inhalation and returns it to the cartridge after use. To ensure dose accuracy, each VC is used by the inhaler only once. In this stability study, only the 500 mcg THC dose was assessed.

### Assessment of pre-packaging MC stability

To assess the effect of refrigeration (5°C±2°C with up to 12% RH) on its stability during the handling and hold time, samples were taken from three separate MC powder batches and assayed at baseline (BL) and at 1, 2, and 3 months after grinding. Each sample was assayed for loss on drying and for the concentration of the major phytocannabinoids (CBDA, CBD, CBGA, CBN, THCA, and THC; ordered according to the ultra-high-performance liquid chromatography (U-HPLC) test).

### Long-term stability of MC stored in cartridges

Once cartridges are produced, they are stored, distributed, and used at room temperature. To assess the shelf life of such cartridges under those conditions, a long-term stability study was performed. To that end, cartridges were stored at 25°C (± 2°C) and relative humidity of 60% (± 5%) in a calibrated and certified stability chamber (Memmert Constant climate chamber HPP260). Batches were tested at BL (*n*=9) and after 3 (*n*=6), 6 (*n*=9), 9 (*n*=8), 12 (*n*=7), 18 (*n*=4), and 24 (*n*=4) months. At each time point, samples of the ground inflorescence within the cartridges were analyzed for loss on drying and for the relative amount (mg) of each of the major phytocannabinoids (CBDA, CBD, CBGA, CBN, THCA, and THC). Cartridges from the same batches were also used for aerosolization by the Syqe inhaler at BL and at 6, 9, 18, and 24 months, and the THC content of the aerosolized dose was analyzed.

To assess the effects of a warmer temperature on MC, cartridges were stored post-production at an elevated temperature of 30°C (±2°C) and relative humidity of 65% (± 5%). Three different batches were analyzed at BL and at 1, 2, 4, and 6 months of storage.

### Chemical analysis of phytocannabinoids

To analyze the six main phytocannabinoids (CBDA, CBD, CBGA, CBN, THCA, and THC) of *Cannabis* inflorescences, 100 mg of ground inflorescences from the same container bulk were weighed in duplicates and combined with 25 mL methanol. The samples were extracted for 40 min in an ultrasonic bath and centrifuged at 3000 rpm for 5 min. After dilution in 70/30 ACN/water + 0.1% formic acid solution, the samples were filtered through a 0.22 μm PP filter vial. The phytocannabinoids were analyzed by an U-HPLC system with a refrigerated auto-sampler, thermostatic column oven, and ultraviolet detector (Waters ACQUITY UPLC H-Class, Waters Corporation, Milford, MA, USA). Chromatographic separation was performed using a Waters ACQUITY UPLC 1.7-μm C18 column 2.1 × 150 mm, maintained at 30°C. The phytocannabinoids were separated using gradient elution with 0.1% (v/v) formic acid in double-distilled water (phase A) and 0.1% (v/v) formic acid in acetonitrile (phase B). A constant flow rate of 0.4 mL/min was employed throughout the analysis. The gradient profile varied from 70% to 100% B in 10.5 min, held for 0.5 min in these conditions, and returned to the initial conditions. The runtime was 14 min. Quantitation of the samples was performed according to calibration curves.

**Fig. 1:**
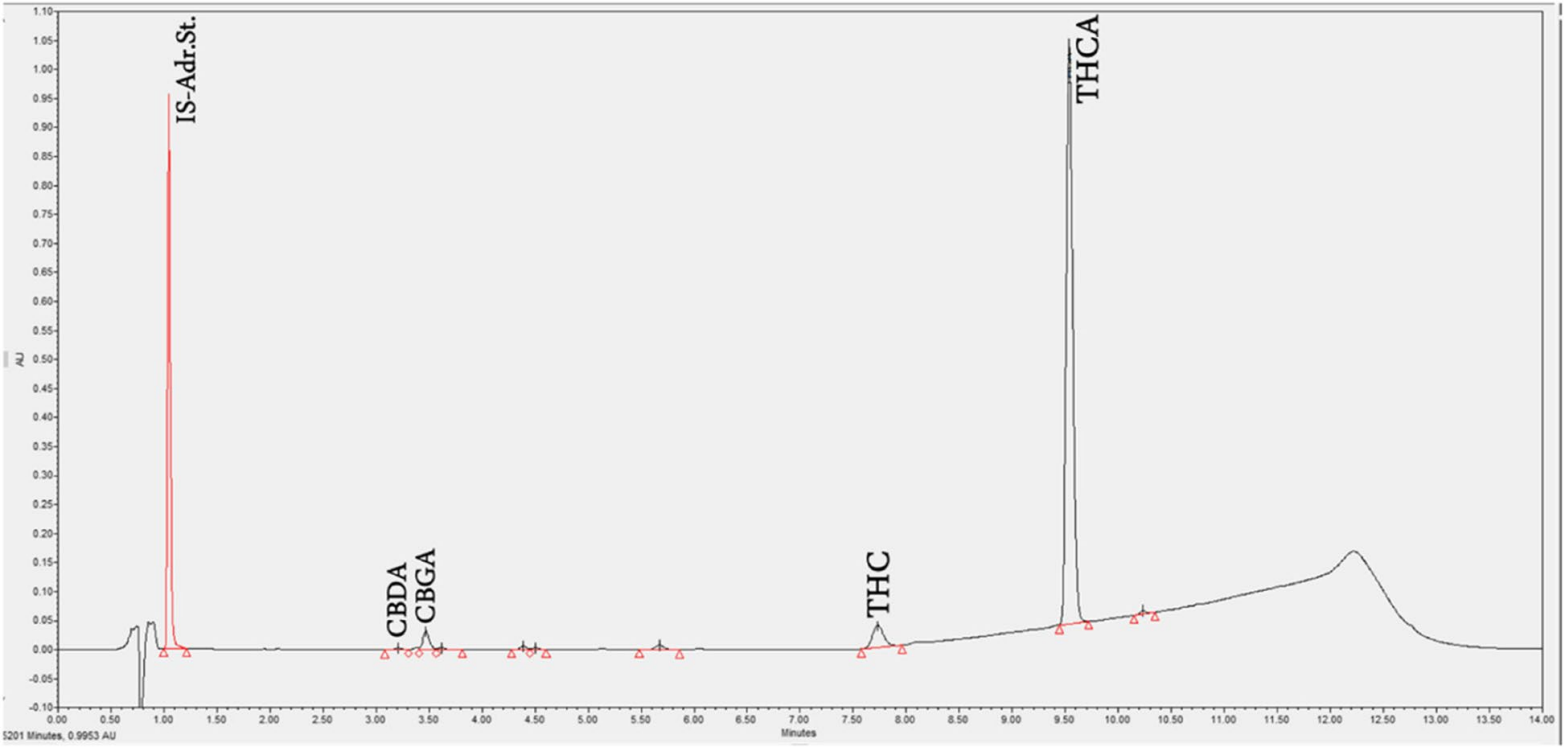
U-HPLC analysis of the Bedrocan MC inflorescence product. From left to right, the analyzed phytocannabinoids were CBDA, CBGA, THC, and THCA. CBN and CBG are undetectable in a fresh specimen. CBDA, cannabidiol acid; CBGA, cannabigerolic acid; THC, (-)-∆^9^-trans- tetrahydrocannabinol; THCA, (-)-∆^9^-trans- tetrahydrocannabinol acid; CBN, cannabinol; CBG, cannabigerol

For THC and CBD, the concentrations of the acid and its neutral counterpart were summed and reported as the total content. For example, the concentration of total THC was calculated as total THC =(THCA ∗ 0.877) + THC, with 0.877 being the molar ratio between the two compounds that correct for a change in the mass of THCA due to the loss of CO_2_ (decarboxylation). Figure 1 is an example of a baseline U-HPLC analysis of the Bedrocan MC inflorescence product.

### Loss on drying

To determine the amount of volatile matter (reported as loss on drying content) in the sample 1.1g ± 0.1g of mixed MC flowers sample or ground powder was weighted and spread in a thin layer over the moisture analyzer (MA100, Sartorius AG, Göttingen, Germany) sample pan. The test was performed in duplicates at 90°C for 10/20 min for ground MC powder or MC flowers raw material, respectively.

### Statistical analysis

The R software package (V.1.1.463) with tidyverse (Tidyverse, 2019) was used to analyze changes in outcome measures by one-way analysis of variance (ANOVA), using Fisher’s exact test. All analyses were followed by a Tukey post hoc test for multiple comparisons. Data are presented as mean and 95% confidence interval (CI). Differences were considered significant at the *p*<0.05 level.

## Results

### The effect of refrigeration on the phytocannabinoid contents of ground *Cannabis* inflorescence prior to packaging

**Fig. 2:**
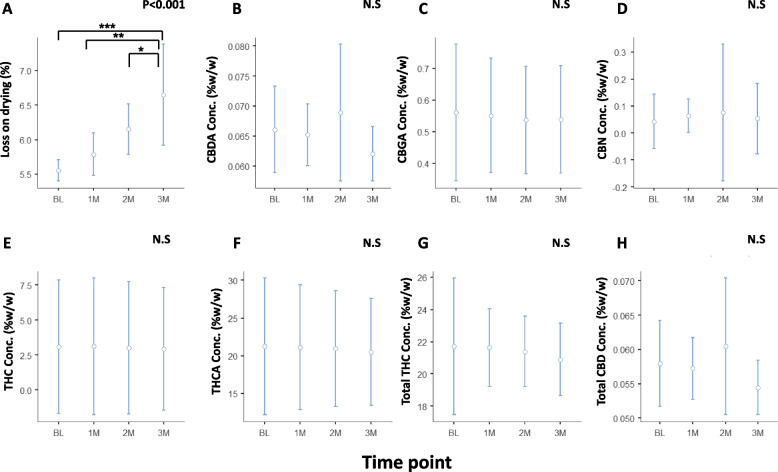
Concentration of major phytocannabinoids in pre-packaged ground *Cannabis* inflorescence stored at 5°C. The middle circle represents the mean value of several duplicates and the upper and lower lines represent the upper and lower 95% confidence interval values. BL, baseline; M, month; CBDA, cannabidiol acid; THC, (-)-∆^9^-trans- tetrahydrocannabinol; THCA, (-)-∆^9^-trans- tetrahydrocannabinol acid; CBGA, cannabigerolic acid; CBN, cannabinol; “Total” represents the sum of the acid and neutral components. Statistically significant differences between times of storage were calculated by one-way ANOVA, utilizing Fisher’s exact test analyses (whenever significant, stated as the *p* value and when not significant stated as N.S.), followed by a Tukey post hoc multiple comparison test (*, *p*<0.05; **, *p*<0.01; ***, *p*<0.001)

A significant increase in loss on drying from 5.6±0.06% at BL to 6.6±0.29% at 3 months (F_(3,8)_=21.56; *p*<0.001) was observed (Fig. 2A). Tukey post hoc test showed a statistically significant difference between loss on drying levels at BL and at 3 months (t_(8)_ = − 7.53; *p*<0.001), at 1 month compared to 3 months (t_(8)_ = − 5.93; *p*<0.01) and at 2 months compared to 3 months (t_(8)_ = − 3.43; *p*<0.05).

As shown in Fig. 2B–H, no significant change was detected during the study in any of the phytocannabinoids: CBDA (F_(3,8)_=2.58, *p*=0.13), CBGA (F_(3,8)_=0.06, *p*=0.97), CBN (F_(3,8)_=0.31, *p*=0.81), THC (F_(3,8)_=0.006, *p*=0.99), THCA (F_(3,8)_=0.03, *p*=0.99), Total THC (F_(3,8)_=0.29, *p*=0.82), and total CBD (F_(3,8)_=2.59, *p*=0.12). As expected, due to the baseline concentrations of CBD in this cultivar (<1%), CBD concentrations were mostly undetected at all time points. These results confirm that the conditions in which the ground inflorescences are preserved maintain stable phytocannabinoid concentrations.

### The effect of storage parameters on phytocannabinoid concentrations in ground *Cannabis* inflorescence packaged in Syqe cartridges

To assess the effect of storage at room temperature on phytocannabinoids content in ground *Cannabis* in Syqe cartridges, a 2-year stability study was performed.

**Fig. 3:**
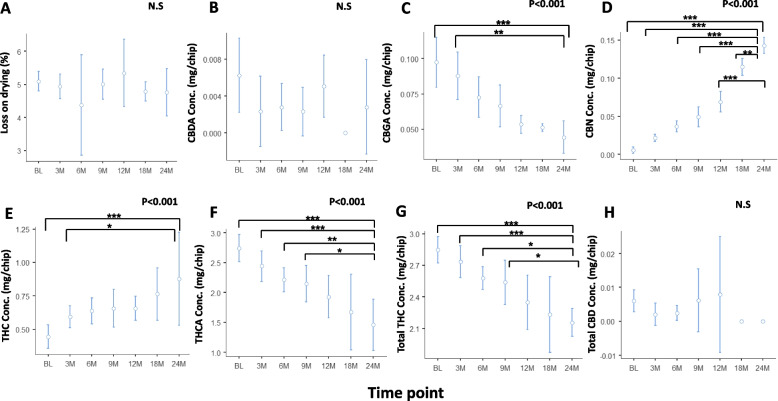
Amount of major phytocannabinoids in MC stored in Syqe cartridges at room temperature over 2 years. The middle circle represents mean value and the upper and lower lines represent the upper and lower 95% confidence interval values. BL, baseline; M, month; CBDA, cannabidiol acid; THC, (-)-∆^9^-trans- tetrahydrocannabinol; THCA, (-)-∆^9^-trans- tetrahydrocannabinol acid; CBGA, cannabigerolic acid; CBN, cannabinol; “Total” represents the sum of the acid and the neutral components. Statistically significant differences between times of storage were calculated by one-way ANOVA, utilizing Fisher’s exact test analyses (whenever significant, stated as the *p* value and when not significant stated as N.S.), followed by a Tukey post hoc multiple comparison test (*, *p*<0.05; **, *p*<0.01; ***, *p*<0.001)

As shown in Fig. 3, no significant changes were observed in loss on drying (F_(6,31)_=0.68, *p*=0.66), in CBDA content (F_(6,40)_=1.90, *p*=0.10) and total CBD content F_(6,37)_=0.67, *p*=0.67). In contrast, significant decreases were observed in CBGA (F_(6,40)_=8.96, *p*<0.001), THCA (F_(6,40)_=11.26, *p*<0.001), and total THC contents (F_(6,40)_=9.94, *p*<0.001). Tukey post hoc test showed a significant decrease in CBGA content between BL and 24 months (t_(40)_=5.33, *p*<0.001) and between 3 months and 24 months (t_(40)_ = − 4.07, *p*<0.01). THCA content decreased significantly between BL and 24 months (t_(40)_=6.72, *p*<0.001), 3 months and 24 months (t_(40)_=4.79, *p*<0.001), 6 months and 24 months (t_(40)_=3.96, *p*<0.01), and between 9 months and 24 months (t_(40)_=3.54, *p*<0.05). Total THC content decreased significantly between BL and 24 months (t_(40)_=5.83, *p*<0.001), 3 months and 24 months (t_(40)_=4.55, *p*<0.001), 6 months and 24 months (t_(40)_=3.56, *p*<0.05), and between 9 months and 24 months (t_(40)_=3.17, *p*<0.05).

CBN and THC content increased significantly (F_(6,40)_=122.18, *p*<0.001 and F_(6,40)_=6.04, *p*<0.001, respectively). Notably, CBN amounts increased between BL and 24 months (t_(40)_ = − 22.18; *p*<0.001), 3 months and 24 months (t_(40)_ = − 18.17, *p*<0.001), 6 months and 24 months (t_(40)_ = − 17.08, *p*<0.001), 9 months and 24 months (t_(40)_ = − 14.82, *p*<0.001), 12 and 24 months (t_(40)_ = − 11.40, *p*<0.001), and between 18 and 24 months (t_(40)_ = − 3.84, *p*<0.01). THC content increased between BL and 24 months (t_(40)_ = − 5.40, *p*<0.001) and between 3 months and 24 months (t_(40)_ = − 3.29. *p*<0.05). As expected, CBD was mostly undetected at all time points, as the pre-storage amount of its precursor, CBDA, was 0.006 mg/sample or less.

**Fig. 4:**
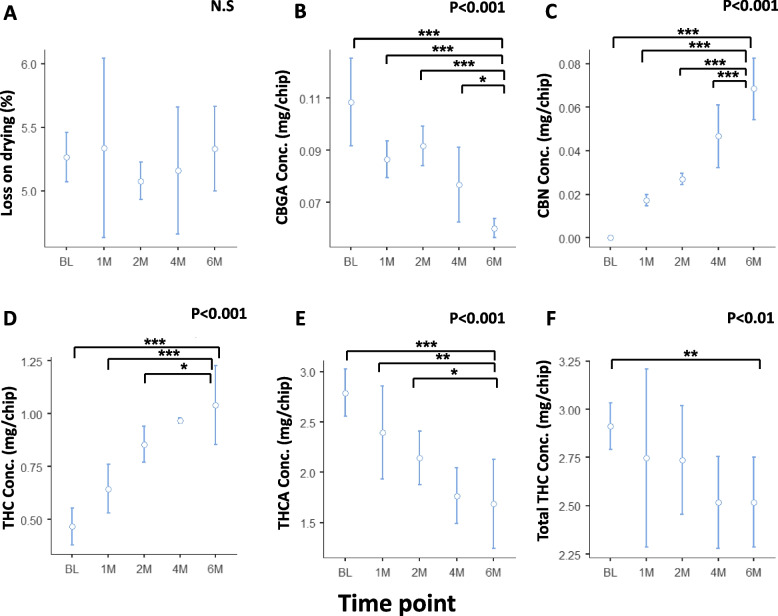
Amount of major phytocannabinoids in MC stored in Syqe cartridges at 30°C over 6 months. The middle circle represents the mean value and the upper and lower lines represent the upper and lower 95% confidence interval values. BL, baseline; M, month; THC, (-)-∆^9^-trans- tetrahydrocannabinol; THCA, (-)-∆^9^-trans- tetrahydrocannabinol acid; CBGA, cannabigerolic acid; CBN, cannabinol; “Total” represents the sum of the acid and the neutral components. Statistically significant differences between times of storage were calculated by one-way ANOVA, utilizing Fisher’s exact test analyses (whenever significant, stated as the *p* value and when not significant stated as N.S), followed by a Tukey post hoc multiple comparison test (*, *p*<0.05; **, *p*<0.01; ***, *p*<0.001)

Analysis of phytocannabinoid content following storage of the Syqe cartridges at an elevated temperature of 30°C, showed that loss on drying values did not vary significantly over time (F_(4,12)_=1.12, *p*=0.19), but CBGA, THCA, and total THC content decreased significantly (F_(4,12)_=33.57, *p*<0.001; F_(4,12)_=22.52, *p*<0.001; and F_(4,12)_=6.46, *p*<0.01, respectively). Tukey post hoc test showed a statistically significant decrease in CBGA content between BL and 6 months (t_(12)_=11.17, *p*<0.001), 1 and 6 months (t_(12)_=5.68, *p*<0.001), 2 and 6 months (t_(12)_=6.76, *p*<0.001), and between 4 and 6 months (t_(12)_=3.55, *p*<0.05). A statistically significant decrease in THCA content was observed between BL and 6 months (t_(12)_=8.39, *p*<0.001), 1 and 6 months (t_(12)_=5.00, *p*<0.01), and between 2 and 6 months (t_(12)_=3.21, *p*<0.05). Total THC content decreased significantly between BL and 6 months (t_(12)_=4.36, *p*<0.01). Additionally, CBN and THC content increased significantly (F_(4,12)_=106.72, *p*<0.001 and F_(4,12)_=44.44, *p*<0.001, respectively). Tukey post hoc test showed a significant increase in CBN amount between BL and 6 months (t_(12)_ = − 19.26, *p*<0.001), 1 and 6 months (t_(12)_ = − 13.30, *p*<0.001), 2 and 6 months (t_(12)_ = − 10.79, *p*<0.001), and between 4 and 6 months (t_(12)_ = − 5.66, *p*<0.001). THC content increased between BL and 6 months (t_(12)_ = − 11.81, *p*<0.001), 1 and 6 months (t_(12)_ = − 7.52, *p*<0.001), and between 2 and 6 months (t_(12)_ = − 3.51, *p*<0.05) (Fig. 4). As expected, CBD, CBDA, and total CBD content were mostly undetected at all time points and could not be analyzed.

### Stability of aerosolized THC dose via the Syqe inhaler over 2 years of cartridges shelf life

**Fig. 5:**
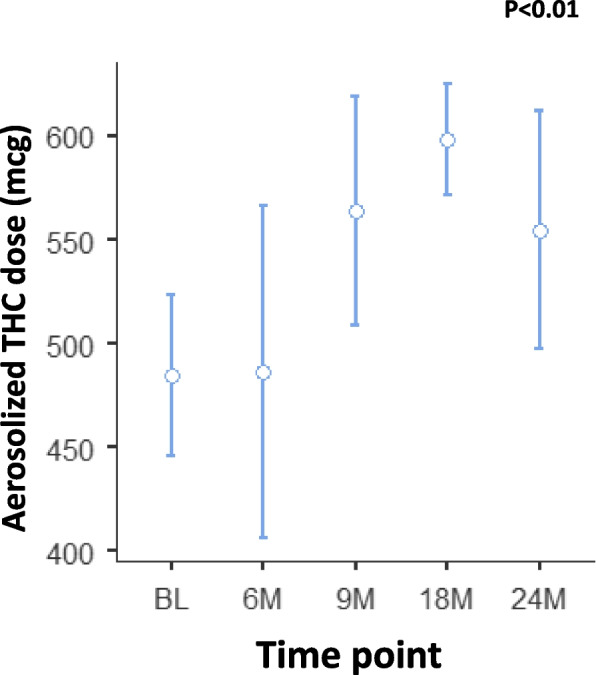
Aerosolized THC doses after storage of MC in Syqe cartridges at room temperature over 2 years. The middle circle represents the mean value and the upper and lower lines represent the upper and lower 95% confidence interval values. BL, baseline; M, month; THC, (-)-∆^9^-trans- tetrahydrocannabinol. Statistically significant differences between times of storage were calculated by one-way ANOVA, utilizing Fisher’s exact test analyses (whenever significant, stated as the *p* value and when not significant stated as N.S.), followed by a Tukey post hoc multiple comparison test (*, *p*<0.05; **, *p*<0.01; ***, *p*<0.001)

In the framework of the 2-year stability study, THC dose was analyzed following aerosolization via the Syqe inhaler. The Syqe inhaler was set to aerosolize 500 mcg THC doses from the standard amount of MC contained within the VCs. Data are shown only for time points in which three or more batches were tested (namely, BL and 6, 9, 18, and 24 months). As shown in Fig. 5, contrary to the changes in the phytocannabinoid amounts in the ground inflorescences inside the cartridges, aerosolized THC doses remained relatively stable, but with some statistically significant changes over time (F_(4,15)_=9.09, *p*<0.001). BL, 6-, 9-, 18-, and 24-months’ values were 484.76±31.28 mcg, 486.30±50.56 mcg, 563±22.06 mcg, 597.94±16.75 mcg, and 554.67±36.00 mcg, respectively. All aerosolized THC doses were well within the 500 mcg ±25% range (i.e., 375–625 mcg) that apply to pharmaceutical-grade inhalers worldwide (18). Tukey’s post hoc test did not show any significant changes between the time points analyzed.

## Discussion

Our analysis did not find significant changes in the main phytocannabinoid concentrations in ground *Cannabis* after 3 months of bulk storage in a polypropylene container and sealed in an aluminum foil pouch at 5°C. This suggests that grinding of the bulk product may be performed at least up to 3 months prior to cartridge manufacture, without a detectable effect on phytocannabinoid composition in ground *Cannabis* inflorescence.

In contrast, significant changes in phytocannabinoid contents were found when the ground inflorescence stored in the cartridges at 25°C over a 2-year period. Specifically, CBGA, THCA and total THC concentrations decreased, while CBN and THC concentrations increased. These findings are in line with those reported by Milay et al. (2020), who reported that a THC-rich cultivar (a cannabis cultivar different than the cultivar of the current study) stored at room temperature for a period of 1 year demonstrated an increase in THC and CBN concentrations, reflecting THC generation due to decarboxylation of THCA by heat and light and simultaneous THC loss by degradation to CBN (Milay et al., 2020). Mentionable, in this comprehensive study, the authors did not perform analyses for THC doses that could be aerosolized at different time points of the analysis. In other words, even though the relative amounts of THCA, THC, and CBN could change during the storage of cannabis, it was not known if this may impact the actual amount of THC that would be released upon aerosolization of these inflorescences.

Although the decrease in THCA and total THC amounts in ground inflorescence stored in the vacuum-sealed cartridges were expected to be correlated with a respective decrease in aerosolized THC doses, these remained within the 500 mcg ±25% range (i.e., 375–625 mcg) as required for pharmaceutical grade inhalers (The United States Pharmacopeial Convention, 2012). This 25% variability range was recommended by the US Pharmacopeial Convention in 2012 for metered-dose inhalers and dry powder inhalers. It should be mentioned that the Syqe inhaler is designed to adhere to pharmaceutical requirement as much as possible in order to provide patients with a consistent aerosolized THC doses that provide consistent THC plasma levels (Eisenberg et al., 2014), and consistent short-term (Almog et al., 2020) and long-term clinical outcomes (Aviram et al., 2022). Syqe has the ambition to become a fully reliable, data-supported, and standardized administration form for inhaled cannabinoids, despite the fact that according to the currently available literature no other MC product currently available in the market follows any clear constraints or requirements on aerosolized THC doses and/or their variability range.

The apparent discrepancy between the decrease in THCA and total THC amounts in ground inflorescence and the relative stability amounts of aerosolized THC doses may be attributed to the increased concentration of THC as well as to the increased proportion of THC/THCA. We hypothesize as follows: The heating process of the Syqe inhaler generates THC-containing aerosol by two mechanisms: decarboxylation (of THCA to generate THC) and vaporization (of THC and THCA). The amount of THC in the aerosol is the sum of vaporized THC and vaporized and decarboxylated THCA. If the efficiency of THC vaporization is greater than that of the vaporization and decarboxylation of THCA then an increase in THC concentration in the plant would more than compensate for an equal loss in THCA concentration. Such overcompensation may explain the observed relative stability of aerosol THC despite a loss of total THC, as observed during storage at 25°C.

Storage of MC in the cartridges at an elevated temperature of 30°C demonstrated a more rapid degradation and decarboxylation process to an extent that would shorten the shelf life of the product. These results show that such temperatures should be avoided during pharmaceutical grade *Cannabis* production, storage, and transport. Furthermore, patients should be encouraged to keep the cartridges at lower temperatures whenever possible.

Our findings suggest that storage at 5°C suffices to maintain phytocannabinoid concentration in MC powder packaged as detailed in this study for up to 3 months. Accordingly, storage by freezing may be unnecessary for this phase of cartridge manufacture, thereby potentially simplifying the logistics of the production process, and reducing its costs. Once a cartridge is produced, it may be kept at room temperature or at a lower temperature to maintain a shelf life of at least 2 years without a significant effect on the dose delivered by aerosolization. Refrigeration may be unnecessary at this stage, potentially simplifying the distribution and storage requirements for the entire supply chain, as well as being more convenient to patients. It is nonetheless important to avoid significant exposure of cartridges to a temperature significantly higher than 25°C, in order to prevent the reduction in phytocannabinoids content.

## Conclusions

Although significant changes in phytocannabinoid amounts occurred during extended storage (up to 2 years) of cartridges containing ground Bedrocan MC inflorescence, the therapeutic aerosolized THC dose remained relatively stable, within the dosage range of 500 mcg ±25% required for pharmaceutical-grade inhalers. This precise aerosolized THC dose, which have yet to be demonstrated in scientific studies by other devices or when *Cannabis* inflorescence is smoked in a cigarette — with or without a tobacco additive — provides an important improvement to the MC field. Future studies should analyze a much wider range of known phytocannabinoids and terpenes present in the *Cannabis* inflorescence and assess the stability of different *Cannabis* cultivars following storage in Syqe cartridges.

## Data Availability

The datasets generated and/or analyzed during the current study are not publicly available due to the intellectual property of Syqe Medical, but are available from the corresponding author on reasonable request.

## Abbreviations

THC: (-)-∆^9^-Trans-tetrahydrocannabinol
THCA: (-)-∆^9^-Trans-tetrahydrocannabinolic acid
CBD: Cannabidiol
CBDA: Cannabidiolic acid
CBNA: Cannabinolic acid
CBN: Cannabinol
U-HPLC: Ultra-high performance liquid chromatography
MC: Medical cannabis
CBGA: Cannabigerolic acid
CBG: Cannabigerol
CBN: Cannabinol
COOH: Carboxyl group
RH: Relative humidity
VC: Vapor chips
RT: Room temperature
CI: Confidence interval
CFU: Colony forming units
Mcg: Microgram
BL: Baseline conditions
